# Sex difference in synaptic plasticity in the anterior cingulate cortex of adult mice

**DOI:** 10.1186/s13041-020-00583-8

**Published:** 2020-03-16

**Authors:** Ren-Hao Liu, Man Xue, Xu-Hui Li, Min Zhuo

**Affiliations:** 1 Center for Neuron and Disease, Frontier Institutes of Science and Technology, Xi’an Jiaotong University, Xi’an, China; 2 Department of Physiology, University of Toronto, 1 King’s College Circle, Toronto, Ontario Canada; 3 Institute for Brain Research, QingDao International Academician Park, Qing Dao, China

**Keywords:** Sex difference, Anterior cingulate cortex, Long-term potentiation, Long-term depression, Female, Male

## Abstract

Sex differences in certain types of pain sensitivity and emotional responses have been previously reported. Synaptic plasticity is a key cellular mechanism for pain perception and emotional regulation, including long-term potentiation (LTP) and long-term depression (LTD). However, it is unclear whether there is a sex difference at synaptic level. Recent studies indicate that excitatory transmission and plasticity in the anterior cingulate cortex (ACC) are critical in chronic pain and pain related emotional responses. In the present study, we used 64-channel multielectrode (MED64) system to record synaptic plasticity in the ACC of male and female adult mice. We found that there was no significant difference in theta-burst stimulation (TBS)-induced LTP between female and male mice. Furthermore, the recruitment of inactive channels was also not different. For LTD, we found that LTD was greater in slices of ACC in male mice than female mice. Our results demonstrate that LTP in the ACC does not show any sex-related difference.

## Introduction

Sex difference in responses to acute and chronic pain have been reported in both animal and human studies. Previous human studies have shown that women have higher sensitivity, lower pain threshold and poorer tolerance for clinical pain than that in the men [1–3]. Recent animal studies have found that female animals showed greater behavioral nociceptive responses to peripheral injury [4, 5]. For example, in the formalin pain model, female mice showed prolonged licking response during late phase of nociceptive response (55–120 min after injection). However, there are also previous reports that showed less sex difference for acute and chronic pain [6, 7].

Anterior cingulate cortex (ACC) is a critical cortical region for pain perception and pain-related emotion [8–10]. The generation and maintenance of chronic pain and pain-related emotions are accompanied by long-term plastic changes or long-term potentiation (LTP) within ACC after peripheral injury. Cortical potentiation induced by the injury contributes to behavioral allodynia, hyperalgesia and spontaneous pain [8, 11, 12]. Synaptic LTP induced by theta-burst stimulation (TBS) is a cellular model for postsynaptic changes in the ACC (or called post-LTP). Animal experiments suggest that, in response to TBS, glutamatergic synapses in the ACC exhibit post-LTP lasting for many hours. However, most of previous studies were carried out in the ACC of adult male mice. Considering possible sex difference in chronic pain, it is important to determine if ACC post-LTP may be different between male and female mice.

In this study we aimed to study the sex difference at synaptic level in the ACC between female and male mice by using a 64-channel multielectrode (MED64) recording system. We analyzed the synaptic plasticity of female and male mice from different aspects. We did not find any significant difference in TBS-induced LTP and recruited synapses between female and male mice. Furthermore, we found that long-term depression (LTD) is greater in slices of ACC in male mice than female mice.

## Methods

### Animals

The animals used in this experiment were adult male and female C57BL/6 mice purchased from the Experimental Animal Center of Xi’an Jiaotong University (8–12 weeks old). All mice used in this experiment were randomly housed under an artificial 12-h light/dark cycle (lights on 9 a.m. - 9 p.m.) with food and water provided ad libitum. Research protocols have been approved by the Ethics Committee of Xi’an Jiaotong University.

### The 64 multi-electrode array

There is an array of 64 square planar microelectrodes (50 × 50 μm/each) arranged in an 8 × 8 pattern in the MED64 probe (P515A, chamber depth 10 mm, Panasonic, Japan), with an interpolar distance of 150 μm. The surface of MED64 probe is relatively hydrophobic. In order to make the slice adhere to MED64 probe well, the new MED64 probe needs hydrophilic treatment. Before experiments, we treated the surface of the MED64 probe with 0.1% polyethyleneimine (Sigma, St. Louis, MO; P-3143) in 25 mmol/L borate buffer (pH 8.4) overnight at room temperature. Then we used sterile distilled water to flush the probe surface three times to remove harmful substances that affect the activity of brain slices [13, 14].

### Brain slice preparation

Acute coronal brain slices (300 μm) containing the ACC were prepared from C57BL/6 mice as previously described [14, 15]. First, we anesthetized mice with 1–2% isoflurane and sacrificed them by decapitation. Then, we quickly removed the entire brain from the skull and submerged it in the ice-cold oxygenated (95% O_2_ and 5% CO_2_) artificial cerebrospinal fluid (ACSF) containing (in mM) 124 NaCl, 2.5 KCl, 2 CaCl_2_, 2 MgSO_4_, 25 NaHCO_3_, 1 NaH_2_PO_4_ and 10 glucose, pH 7.3–7.4. After a brief cooling, the brain was cut into coronal brain slices (300 μm) containing the ACC and the slices were transferred to a submerged recovery chamber with oxygenated (95% O_2_ and 5% CO_2_) ACSF at room temperature for at least 1.5 h.

### Field potential recording

**Fig. 1:**
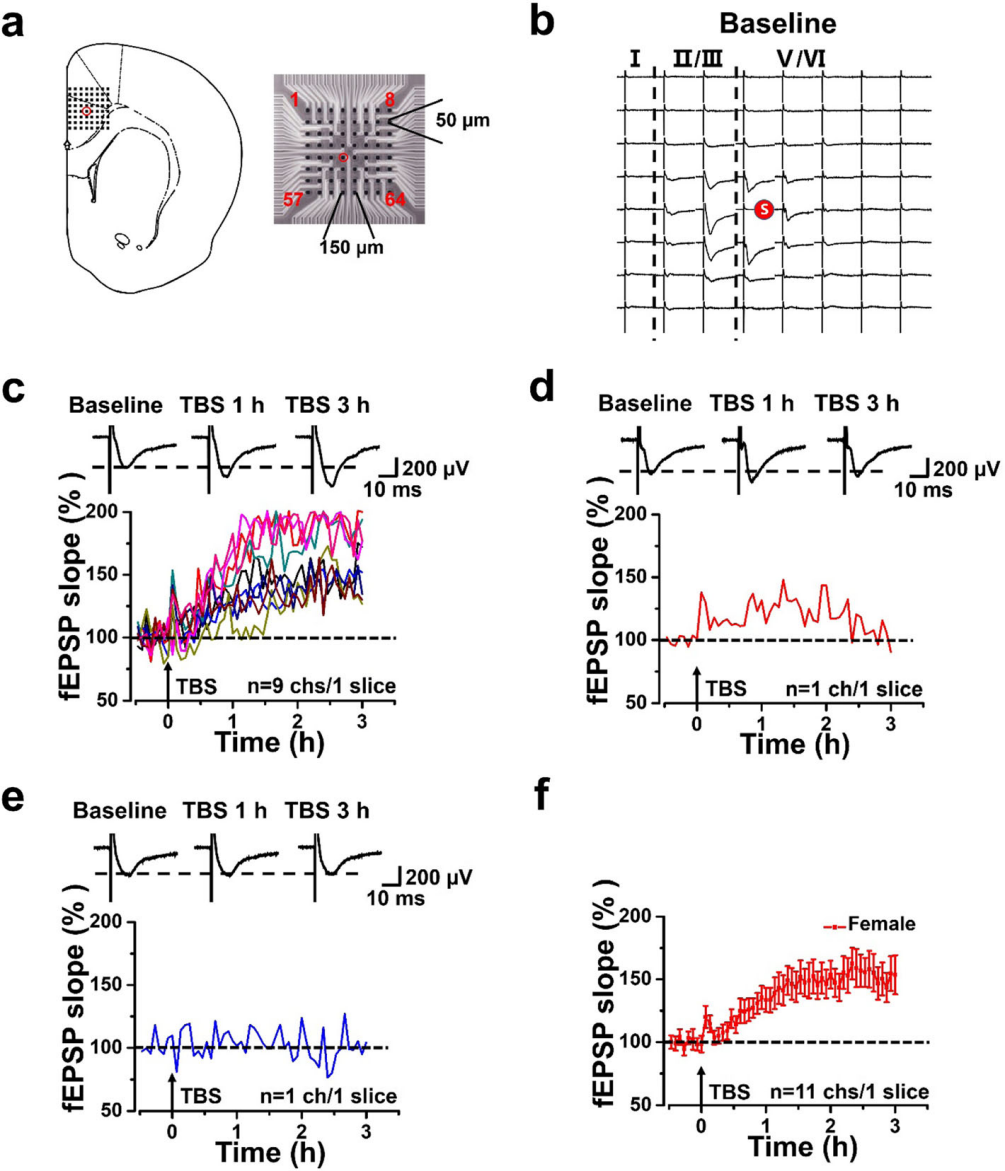
LTP recorded from a slice of female mouse by extracellular field potential recording. **a** Schematic diagram of stimulation site (red circle) of microelectrodes in the ACC and the arrangement of the microelectrodes (electrode size 50 × 50 μm, interpolar distance of electrodes 150 μm). **b** Spatial distribution of extracellular field potential induced by electrical stimulation on channel 36 (marked as red circle) in layers V of the ACC. **c-e** Time-varying fEPSP slope in all activated channels from one slice in female mouse: 9 channels with late phase LTP (L-LTP) (**c**), 1 channel with short-term potentiation (E-LTP) (**d**), and 1 channel without potentiation (none-LTP) (**e**). **f** The final averaged slope of all 11 channels at 3 h after TBS from one slice of the female mouse (152.36 ± 13.81% of the baseline)

We performed the MED64 recording system (Panasonic, Japan) to record extracellular field potential in the ACC in female and male adult mice. After incubation, one slice containing ACC was transferred to the prepared recording chamber and perfused with oxygenated (95% O_2_ and 5% CO_2_) ACSF at 28–30 °C and maintained at a 2 ml/min flow rate. The slices were placed on the MED64 probe, so that the whole microelectrode array can cover different layers of the ACC, and the middle part of the probe is close to the central point of ACC. Then a channel located in the layer V of ACC was selected as the stimulation site, which can induce the best synaptic responses in the surrounding recording channels from deep to superficial layers. Figure 1a showed a sample of an ACC slice placed on a MED64 probe. Before carrying out experiment, the slices were kept in the probe at least 1 h. Bipolar constant current pulse stimulation (1–10 μA, 0.2 ms) was applied to the stimulation site and the intensity was adjusted so that we can evoke a half-maximum field excitatory postsynaptic potential (fEPSP) in the channel nearest to the stimulation site. The channel with fEPSP was regarded as an activated channel and its fEPSP response was sampled every 1 min and averaged every 2 or 4 min. The ‘slope’ parameter represented the average slope of each fEPSP recorded by the activated channel. Stable baseline responses (variation in the baseline response of a single channel is < 5% and the number of channels with unstable baseline responses was ≤10% of the total number of active channels) were first recorded for 30 min. Then, a TBS (five trains of bursts with four pulses at 100 Hz at 200 ms interval; repeated five times at intervals of 10 s) with the same intensity as the baseline stimulation was applied to the same stimulation channel to induce LTP. After induction of LTP, the fEPSP responses were recorded for 3 h.

For LTD induction, a stable baseline (same as LTP recording) was recorded for 15 min and then a classical low-frequency stimulation (LFS) protocol (1 Hz, 900 pulses, with the same intensity as baseline recording) was used as described previously [16, 17]. If the number of unstable channels was > 10%, the slice would be discarded. The fEPSP responses were recorded for 30 min after applying LFS on stimulation site.

### Data analysis

The data presented as means ± SEM. Statistical comparisons between two groups were performed using two-tail unpaired Student’s *t*-test, one-way ANOVA to identify significant differences. In all cases, * *P* < 0.05 was considered statistically significant.

## Results

### LTP induced by TBS in the ACC of female mice

LTP plays an important role in cortical excitation in chronic pain [8, 18, 19]. In our previous work, the intercellular network plasticity in the ACC was studied focuses on the adult male mice, and the LTP induced by TBS was mainly recorded in the ACC of male mice by MED64 recording system [17, 20, 21]. Here we performed the same protocol to study the network plasticity in the ACC of adult female mice. We placed a slice containing the ACC of female mice on the MED64 chamber and ensured the recording microelectrodes covered the ACC area (Fig. 1a). One channel located in the deep layer of ACC was chosen as stimulation site (Fig. 1a, red circle). The fEPSP slope of all activated channels were recorded from superficial to deep layer around the stimulation site for 30 min as a baseline (Fig. 1b). After recording 0.5 h stable baseline, we applied TBS to evoke network LTP in the ACC. Consistent with previous reports from male mice, we found that TBS induced L-LTP (late-phase LTP) in the ACC of female mice (*n* = 14 slices/9 female mice, Fig. 1c-e). In one typical female sample slice, there were 11 activated channels, among which there were 9 channels showed L-LTP (165.2 ± 11.6% of the baseline, Fig. 1c) and 1 channel showed E-LTP (early-phase LTP) (Fig. 1d), while 1 channel showed none-LTP (Fig. 1e). The final averaged slope of all 11 channels from the typical sample slice of the female mouse was 152.4 ± 13.8% of the baseline at 3 h after applying TBS (Fig. 1f). These results are similar with that in our previous work performed in male mice [14, 20].

### No sex difference of LTP in the ACC between adult female and male mice

**Fig. 2:**
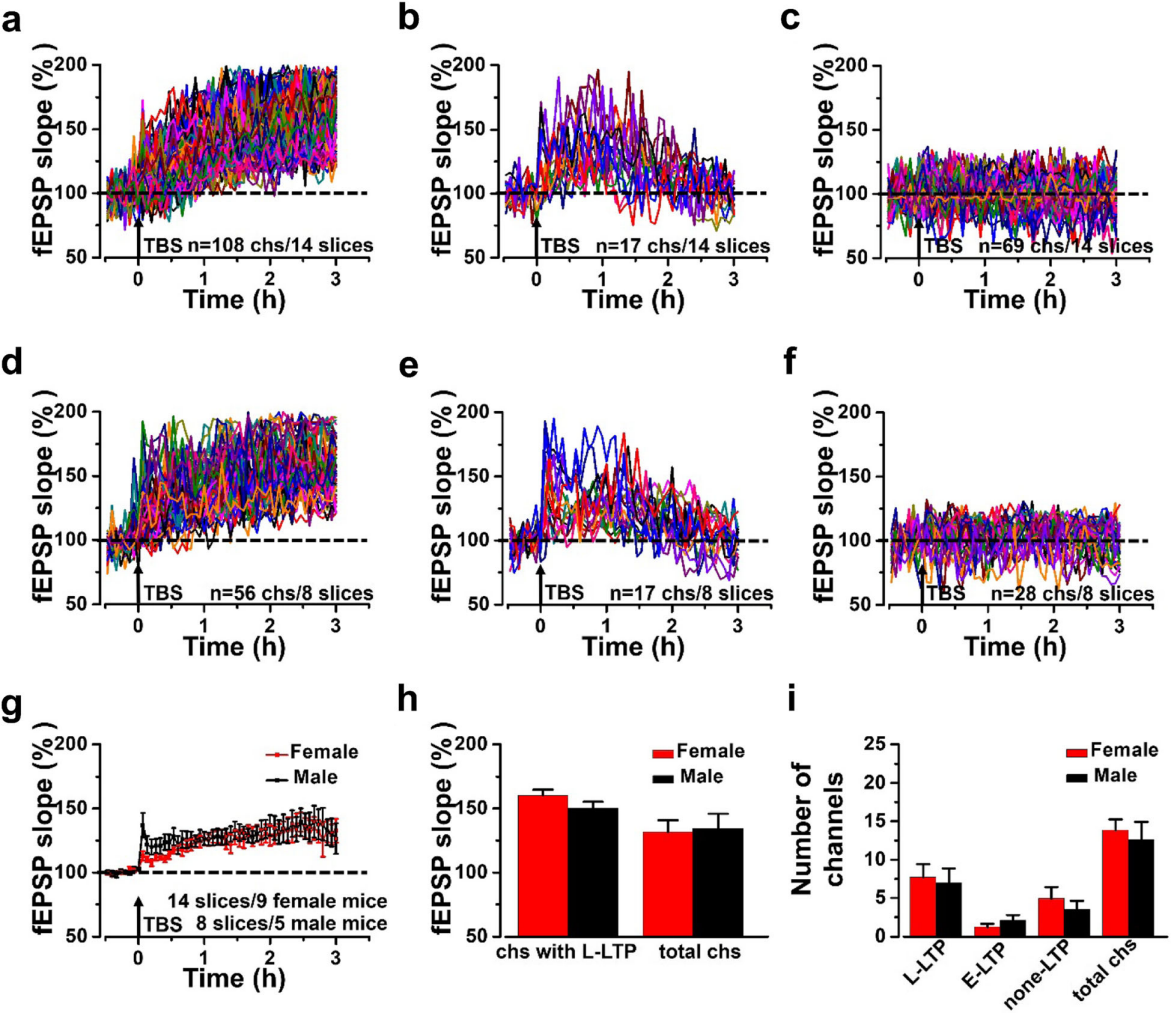
Network plasticity of the fEPSPs after LTP induction in the ACC of female and male mice. **a-c** All channels with L-LTP (**a**), E-LTP (**b**) and none-LTP (**c**) were induced in female mice (14 slices/9 mice). **d-f** All channels with L-LTP (**d**), E-LTP (**e**) and none-LTP (**f**) were induced in male mice (8 slices/5 mice). **g** Time-varying fEPSP slope of all recording channels were summarized in female and male mice. **h** No difference in averaged fEPSP slope of all channels with L-LTP and all activated channels between female and male mice. **i** No difference of average number of three different types of plasticity between female and male mice

To investigate whether there is any sex difference in LTP within ACC between male and female mice, we recorded LTP in both female and male mice by performing MED64 recording system. In all 194 activated channels that we recorded from 14 slices from 9 female mice, we found that there were 108 channels with L-LTP, 17 channels with E-LTP and 69 channels showed none-LTP and the induction rates of three different types of channels were 54.9 ± 10.4%, 10.3 ± 4.0% and 34.8 ± 9.2%, respectively (Fig. 2a-c). In the male mice, there were 101 channels activated from 8 slices in 5 male mice. We found there were 56 channels with L-LTP, 17 channels with E-LTP and 28 channels showed none-LTP and the induction rates of three different types of channels were 52.8 ± 14.2%, 22.1 ± 7.8% and 25.1 ± 9.9%, respectively (Fig. 2d-f).

The final averaged fEPSP slope of all activated channels was 131.6 ± 9.3% of the baseline at 3 h in female mice and 134.5 ± 11.5% of the baseline at 3 h in male mice (Fig. 2g and h). Meanwhile, the final averaged fEPSP slope of all channels with L-LTP was 160.4 ± 4.4% of the baseline at 3 h in female mice and 150.4 ± 5.1% of the baseline at 3 h in male mice (Fig. 2h). There was no difference in fEPSP slope either in all activated channels or channels with L-LTP between the female mice and male mice. Further analysis of the number of channels with L-LTP, E-LTP and none-LTP respectively from 14 slices from 9 female mice showed that there were 7.3 ± 1.6 channels with L-LTP, 1.6 ± 0.4 channels with E-LTP, and 2.0 ± 1.7 channels with none-LTP in each slice of female mice on average (Fig. 2i). In male mice, there were 7.0 ± 1.9 channels with L-LTP, 3.8 ± 1.6 channels with E-LTP and 3.5 ± 1.1 channels with none-LTP in each slice of male mice on average (Fig. 2i). There was no statistical difference in the number of different types of channels between female and male mice. Taken together, our results suggested that there was no sex difference of LTP in the ACC between female and male adult mice.

### Silent synapses were recruited after the induction of LTP in the ACC of female and male mice

**Fig. 3:**
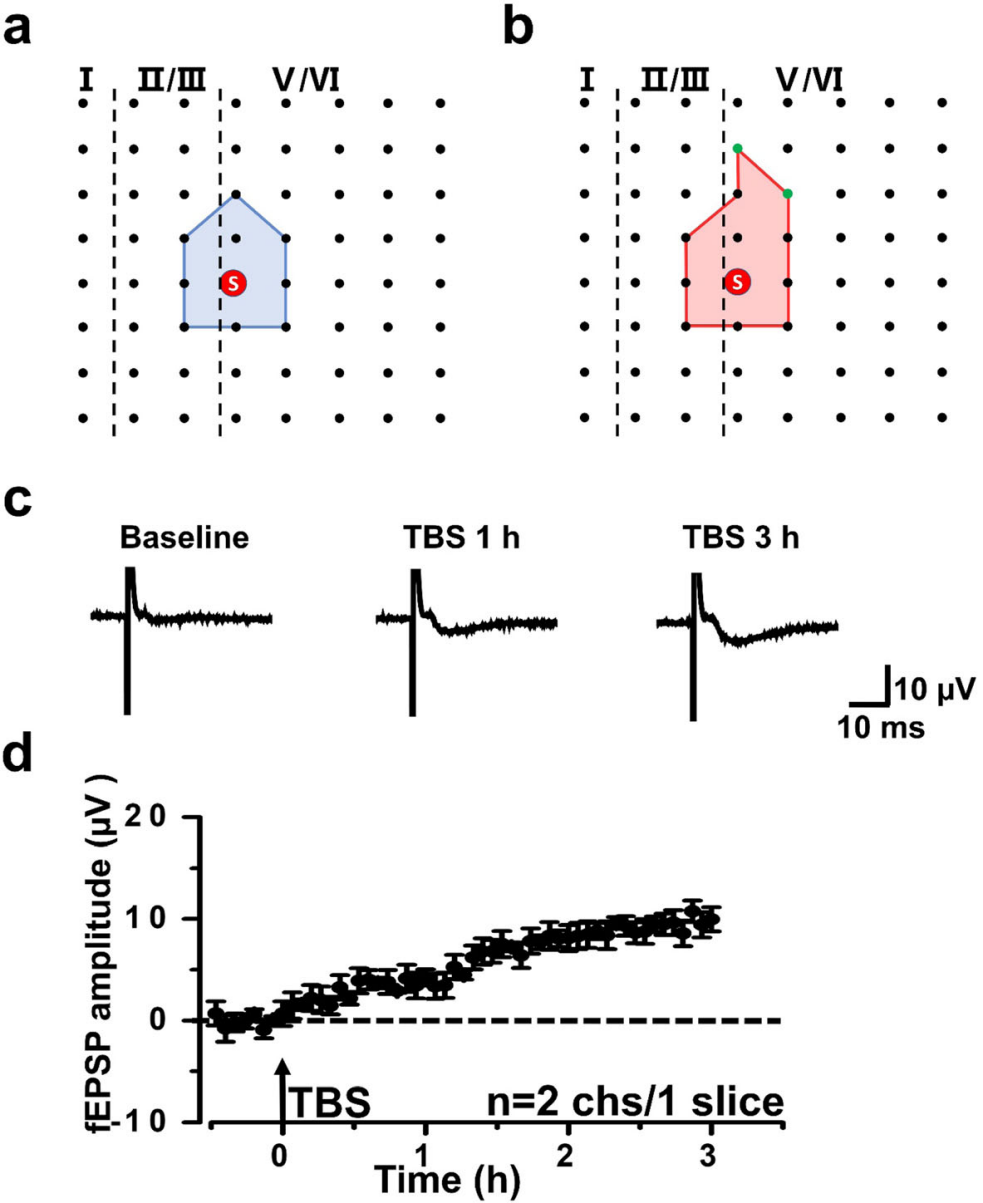
Recruited responses induced by TBS in the ACC of a female mouse. **a**, **b** Basal activated area (blue) and recruited area (red) induced by TBS in a typical slice from female mouse. The recruited channels were shown as green dots. **c** The sample traces indicated one channel showing recruited fEPSPs after TBS. **d** Time-varying fEPSP amplitude in two typical recruited channels from one female slice

Previous studies showed that some silent responses were recruited after L-LTP induction. To determine whether there was any sex difference in recruited synapses between female and male mice, we analyzed all recruited channels from female and male mice, respectively. In Fig. 3, it showed a spatial distribution and the change of fEPSP amplitude of recruited channels in a typical slice of female mice. The recruited channels mainly located on the edge of the activated area of female mice (Fig. 3a and b) and the amplitude which was about 0 μV during baseline would increase over time after applying TBS protocol (Fig. 3c and d).

**Fig. 4:**
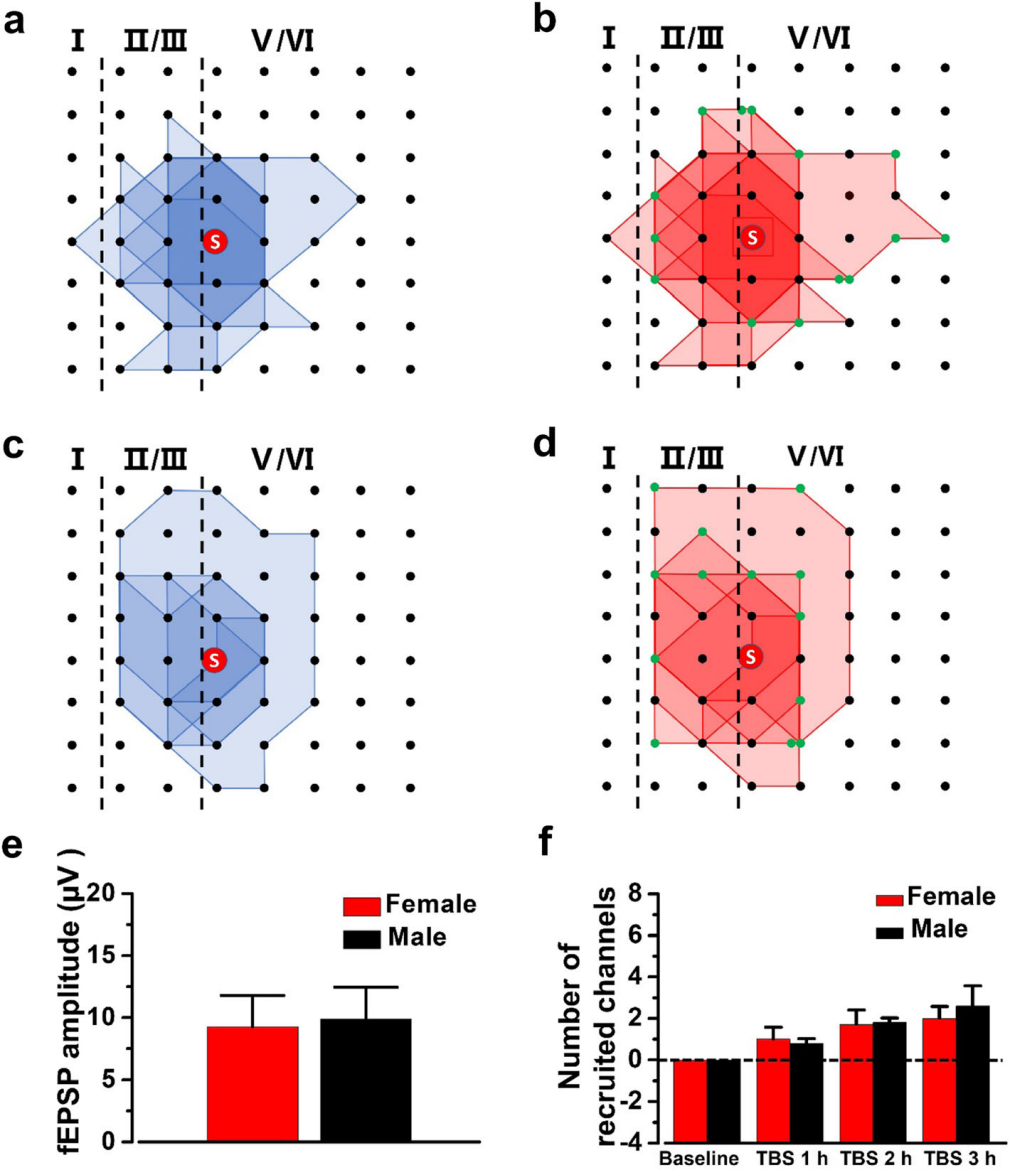
The network propagation of synaptic responses in the ACC of female and male mice. **a**-**d** Basal activated areas (blue) and recruited areas (red) induced by TBS in female (**a** and **b**) and male (**c** and **d**) mice, respectively. The recruited channels are shown as green dots. **e** The averaged amplitude of the recruited fEPSPs at 3 h after TBS in female and male mice. **f** The temporal changes of the average number of the recruited channels in female and male mice. *n* = 7 slices from 6 female mice and *n* = 5 slices from 3 male mice, respectively

Only a portion of slices would successfully recruit silent channels after L-LTP induction. Therefore, in the Fig. 4, the spatial distribution of all recruited channels was showed from 7 slices of 6 female mice and 5 slices of 3 male mice. In both of female and male mice, the recruited channels were mainly located on the edge of the basal active area (Fig. 4a-d). Then, we also did an in-depth analysis of the number and fEPSP amplitude of recruited channels to see whether there was any sex difference between female and male mice. The final averaged fEPSP amplitude of all recruited channels was 9.3 ± 2.5 μV and 9.9 ± 2.6 μV of the baseline at 3 h in female mice and male mice, respectively (Fig. 4e). There was no statistical difference in the fEPSP amplitude of recruited channels between female and male mice. After L-LTP induction, 2.0 ± 0.6 channels would be recruited at 3 h in each slice of female mice on average which was similar to that in male mice (2.6 ± 1.0 recruited channels, Fig. 4f). These results showed that both the recruited fEPSP amplitude and the number of the recruited channels had no general difference in female and male mice.

### LTD in male and female mice

**Fig. 5:**
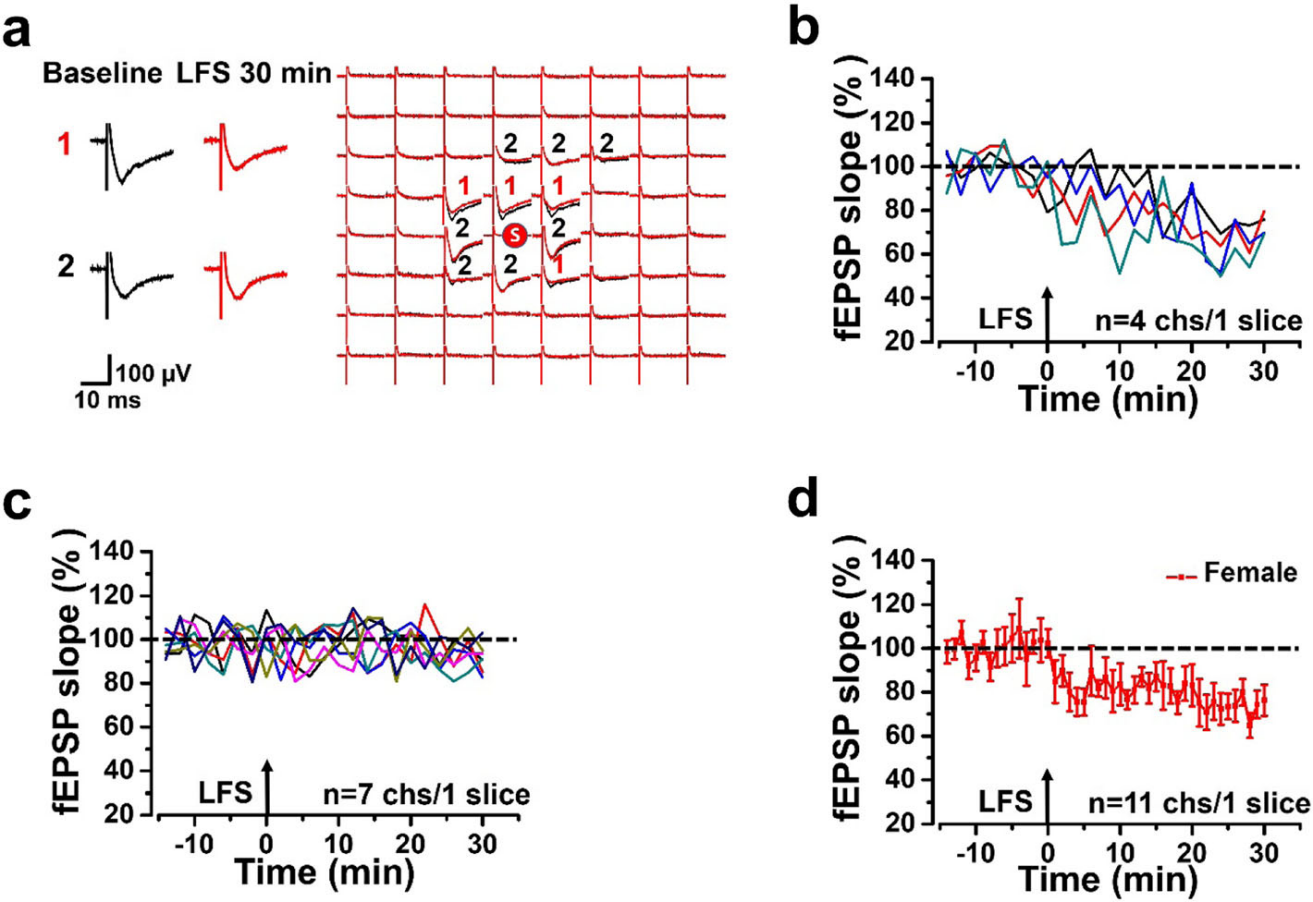
Recording LTD in the ACC of a female mouse. **a** The spatial distribution of evoked field potentials in the female mouse ACC of baseline (black) and the variation of fEPSPs after LFS (red). The superimposed sample on the left side of the network figure showed two different types of plasticity: channels with a slope decrease of more than 20% represent LTD marked as ‘1’; channels that the fEPSP slope don’t change after LFS are marked as ‘2’. **b**-**c** Time-varying slope in all activated channels from one slice of the female mouse: 4 channels with LTD (**b**) and 7 channels with none-LTD (**c**). **d** The final averaged slope of all 11 channels after LFS (83.30 ± 7.63% of the baseline) from one slice of female mouse

LTD plays an important physiological role in pain by weakening the synaptic connections [21, 22]. In order to study possible sex-related difference in LTD, we recorded LTD in the ACC of adult female and male mice. We recorded the fEPSPs baseline of all activated channels for 15 min before LTD inducing protocol wad applied (LFS: 1 Hz, 900 pulses, with the same intensity as baseline recording). As shown in Fig. 5, we got two different types of synaptic plasticity in all activated channels: channels with a slope decrease of more than 20% at 30 min represented LTD and channels that the fEPSP slope did not change after LFS represented none-LTD (Fig. 5a). In a typical sample slice of a female mouse, there were 11 channels being activated, including 4 channels with LTD and 7 channels with none-LTD after LFS (Fig. 5b and c). The final averaged slope of all 11 channels from the typical female sample slice was 83.3 ± 7.6% of the baseline at 30 min after applying LFS (Fig. 5d).

**Fig. 6:**
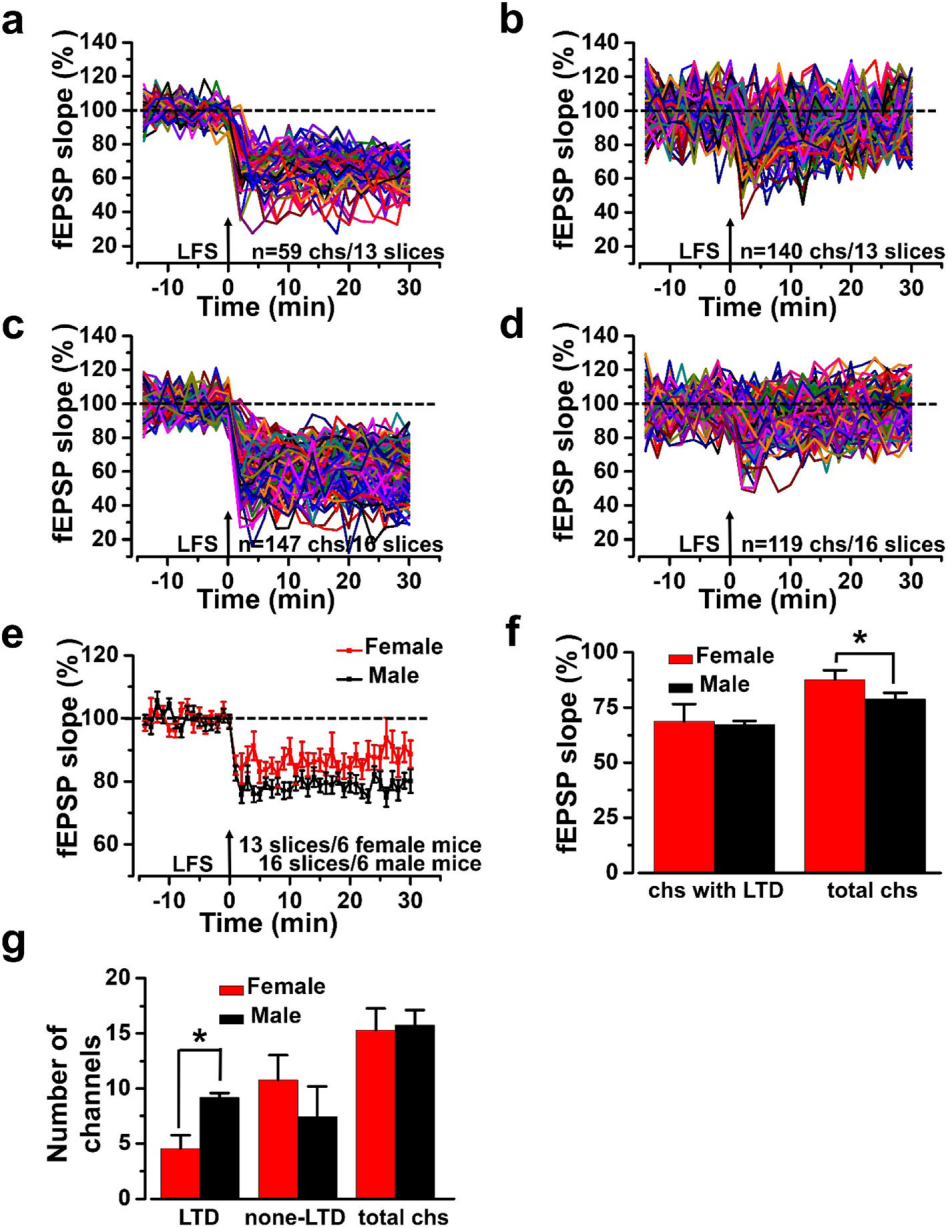
LTD induced by LFS in the ACC of male and female mice. **a**, **b** All channels with LTD (a) and none-LTD (b) in female mice. **c**, **d** All channels with LTD (**c**) and none-LTD (**d**) in male mice. **e** Time-varying fEPSP slope of all recording channels from female (13 slices/6 mice) and male (16 slices/6 mice) mice. **f** The averaged fEPSP slope of all activated channels with LTD and all activated channels from female and male mice. **g** The average number of channels of two different types of synaptic plasticity from female and male mice. * *P* < 0.05, female mice vs male mice

Next, we compared the sex difference of LTD in female and male mice. There were 199 activated channels from 13 slices of 6 female mice and 266 activated channels from 16 slices of 6 male mice, respectively. In these activated channels, the induction rate of channels with LTD was 33.7 ± 8.5% in female mice and 53.5 ± 7.7 in male mice (*P <* 0.05, Fig. 6a-d). Although there was no difference in the final averaged slope of all channels with LTD between male and female mice after applying LFS on stimulation site (Fig. 6f), the final averaged slope of all activated channels from 16 slices of 6 male mice (78.8 ± 2.8% of the baseline) was not as high as that from 13 slices of 6 female mice (87.6 ± 4.3% of the baseline, *P <* 0.05, female mice vs male mice, Fig. 6e and f).

In addition, we found that when there was no difference in the total number of activated channels between female and male mice, the number of channels with LTD in each slice of male mice on average (9.2 ± 0.4 channels) was significantly higher than that in the female mice (4.5 ± 1.2 channels, *P <* 0.05, Fig. 6g). These results suggested that LFS was more likely to induce LTD in the ACC of male mice than that in female mice. This difference was mainly concluded from the number of channels with LTD.

### Silent synapses appeared in the ACC of adult mice after LFS

**Fig. 7:**
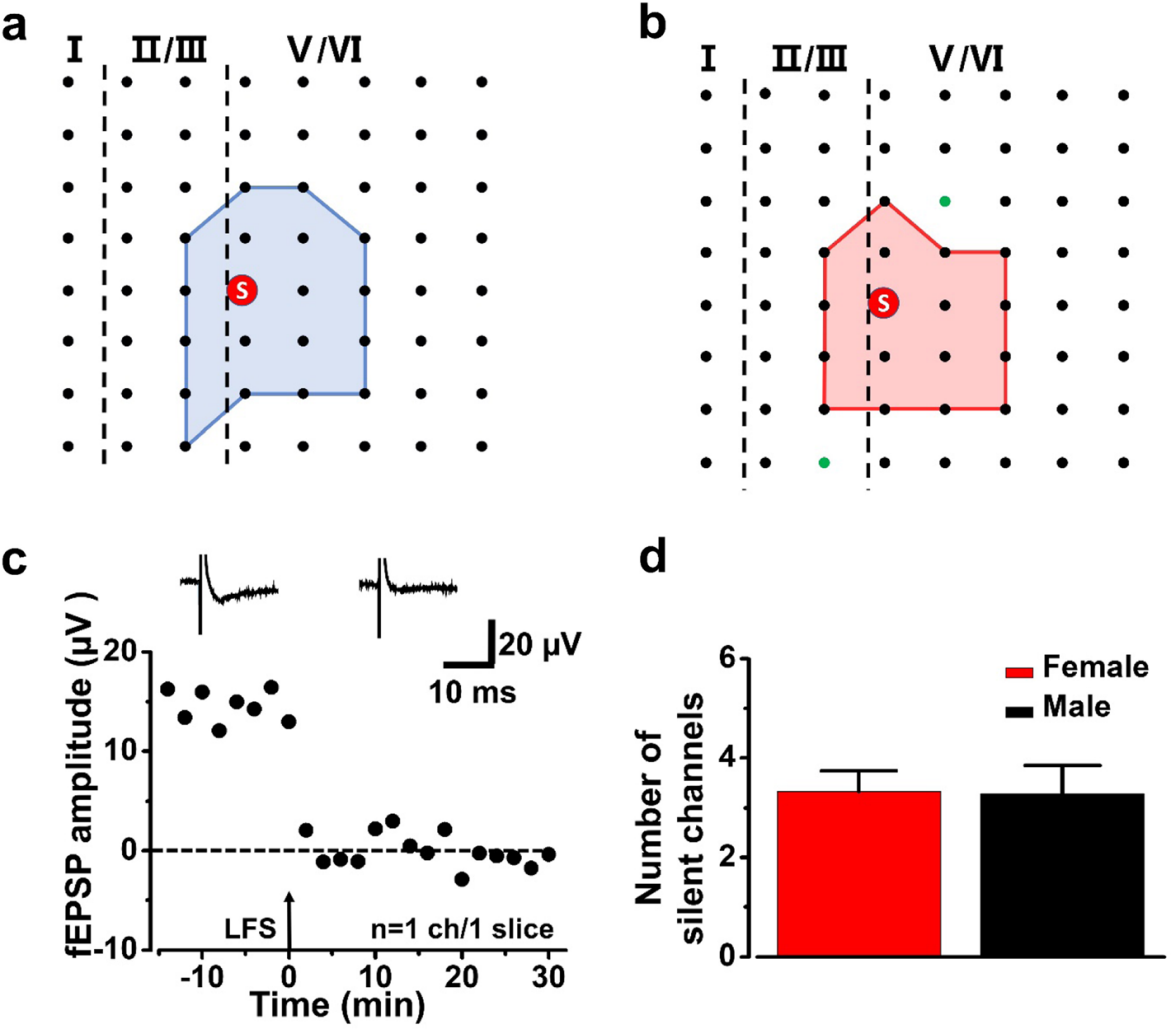
Activated channels were silenced by LFS in female and male mice. **a**, **b** Basal activated area (blue) and silent area (red) induced by LFS in a typical slice from a female mouse. The silent channels are shown as green dots. **c** The change of fEPSP amplitude of the silent channel in a typical slice from a female mouse after LFS. **d** The average number of the silent channels in female and male mice in one slice

There are hypotheses indicate that functional synapses can be transformed into silent synapses by endocytosis and inactivation of postsynaptic membrane α-amino-3-hydroxy-5-methyl-4-isoxazolepropionic acid (AMPA) receptor [23–26]. In the present study, we found that some of active channels became silent after LFS. As shown in Fig. 7a and b, the activated area was reduced after LFS in one typical slice from female mice. At 30 min after LFS, the amplitudes of the channels that were activated during baseline were decreased to about 0 μV (Fig. 7c). Next, we compared the number of silent channels that appeared after LFS in the ACC between male and female mice. There are 3.3 ± 1.5 silent channels in each slice of female mice on average and this result was similar to that in male mice (3.3 ± 0.8 silent channels, Fig. 7d).

## Discussion

Synaptic plasticity within ACC and insular cortex (IC) is a key cellular mechanism for chronic pain [8, 9]. Most of previous studies were carried out in adult male mice and these results are not able to explain the increasing reports of sex differences in pain. In the present study, we compared the sex differences at synaptic plasticity level between female and male mice in the ACC. We found that there was no sex difference in post-LTP between female and male mice. For LTD, we found that in comparison with female ACC, the number of channels with LTD was significantly higher in male mice than that in female mice.

In our present work, we compared the characteristics of LTP in the female and male mice. We found that TBS can induce similar LTP in female as in male mice. Furthermore, both percentage of channels that undergoing LTP and the magnitude of LTP are similar between male and female mice. Our LTP results from male mice are similar to LTP reported in our previous reports [14, 21]. These results clearly indicate that there is no significant difference in LTP in the ACC between male and female adult mice. However, in other brain areas such like hippocampus and amygdala, sex-related differences in LTP have been reported, although some results are contradictory [27–30]. For example, Chen et al. showed that female mice had a greater LTP magnitude in amygdala as compared to that in male mice [29]. But Bender et al. found that there was no sex difference for LTP in amygdala [30]. These inconsistent results of sex differences in synaptic plasticity may be due to the different regions of the brain and various induction paradigm employed.

Another similarity between male and female mice is the recruitment of silent synapses after the induction of L-LTP [14, 20]. Our recent study reported that phosphorylation at s845 site of glutamate receptor 1 (GluA1) and possible postsynaptic trafficking of AMPA receptor may contribute to the recruitment of silent response [14, 31, 32]. In addition to N-methyl-D-aspartate (NMDA) receptor dependent post-LTP we reported in this study, there are other forms of LTP in the ACC [8, 9, 33]. We cannot rule out possible differences in other forms of LTP such as NMDAR independent LTPs [33, 34] between male and female mice. Future studies are clearly needed.

LTD is another kind of synaptic plasticity in the ACC. In the present study, we found that the amplitude of LTD is similar between female and male mice. However, the number of LTD expression sites in male mice was significantly higher than that in female mice, indicating there is a sex difference in network plasticity between female and male mice. This implies that female and male mice may have sex difference in the development of ACC structure. The sex difference in the structure and function of ACC is also confirmed by functional magnetic resonance imaging (fMRI). In the control of negative emotion, there was a significantly stronger activity in the areas of cognitive control in men, including ACC area [35, 36].

Altered synaptic responses and synaptic plasticity including LTP and LTD have been thought to contribute to ACC related chronic pain and fear conditioning [9, 29, 37]. In rodent models of chronic pain, previous studies demonstrated that synaptic responses in the ACC were potentiated and LTP was occluded or impaired. Inhibition of the induction of LTP or reducing the expression of LTP can produce analgesic effects in animal models of chronic pain [8, 9, 12]. In addition to chronic pain, ACC has also been indicated in fear memory. Fear conditioning potentiated synaptic transmission in the ACC, and inhibiting excitatory transmission or the induction of LTP reduce fear [37–40]. Female rats generalize contextual fear at a faster rate than males and this effect is due to estradiol [41]. Estradiol may affect fear memory through AMPA receptor and NMDA NR2B receptors [42]. Future studies are clearly needed to examine possible sex difference in central excitatory synapses in the ACC and its related areas.

In our present work, we found that there was no sex difference in LTP in ACC. As a crucial region for chronic pain and fear memory, the result of no sex difference in LTP in ACC demonstrate that sex difference in chronic pain might be related to other mechanisms. A recent study reported that ACC could exert a top-down facilitatory modulation for spinal nociceptive transmission [43]. Future studies are clearly needed if such top-down modulatory system may also show sex-related difference. In addition to LTP, LTD is another key form of plasticity in the ACC. Previous studies indicated that the induction of LTD was affected after peripheral tissue injury [16, 44, 45]. For example, activation of mGluA1 rescues LTD impairment in ACC caused by tail amputation [16]. In a mouse model of bone-cancer pain, LTD in the ACC is also impaired [44]. Most of previous reports were collected from adult male mice, less studies were performed in the ACC of female mice. In addition, there is less study about LTD in ACC under fear conditioning. A recent study showed that chronic restraint stress reduced freezing behavior, and enhanced LTD in ACC [46]. Future studies are needed to investigate sex-related difference in ACC LTD under different physiological and pathological conditions.

In summary, our results demonstrated that there was no sex difference in LTP in ACC, but the expression of LTD in male mice was slightly higher than that in female mice. Previous studies have shown that chronic pain is related to the saturated late component of LTP and the inhibition of LTD in ACC [11, 13, 16, 44]. Therefore, our study indicates that sex related difference in LTD may contribute to sex difference in persistent or chronic pain, although we cannot rule out the possible difference in other forms of LTP.

## Data Availability

The datasets used and/or analysed during the current study are available from the corresponding author on reasonable request.

## Abbreviations

ACC: anterior cingulate cortex
ACSF: artificial cerebrospinal fluid
AMPA: α-amino-3-hydroxy-5-methyl-4-isoxazolepropionic acid
ANOVA: analysis of variance
CRS: chronic restraint stress
fEPSP: field excitatory postsynaptic potential
fMRI: functional magnetic resonance imaging
GluA1: glutamate receptor 1
LFS: low-frequency stimulation
LTD: long-term depression
LTP: long-term potentiation
MED64: 64-channel multielectrode
NMDA: N-methyl-D-aspartate
SEM: standard error of the mean
TBS: theta-burst stimulation
